# A Hypothesis Testing of Archwire Rounding for the Efficacy of Torque Springs in Orthodontics: A Finite Element Study

**DOI:** 10.7759/cureus.57292

**Published:** 2024-03-30

**Authors:** Blend H Balisany, Omar F Chawshli, Zana Q Omer

**Affiliations:** 1 Pedodontics, Orthodontics and Preventive Dentistry, College of Dentistry, Hawler Medical University, Erbil, IRQ

**Keywords:** fea, warren torque spring, torque, finite element method, orthodontics

## Abstract

Background: Achieving the proper buccolingual inclination of teeth is a cornerstone in orthodontic treatment, directly impacting the attainment of ideal occlusal relationships and long-term stability. A practical torque expression that moves the tooth in its proper position across all three planes is imperative to finish orthodontic cases optimally. The primary focus of this research is to investigate Burstone's hypothesis about Warren torque springs when applied to the rectangular wire. Additionally, it examines the hypothesis of rounding these wires in between the bracket wings of the target tooth to be moved. This study aims to determine whether the rounding of wires, in conjunction with the use of torque springs, influences orthodontic outcomes, addressing a notable gap in current literature and resolving controversies in orthodontic practice.

Methods: A three-dimensional set of maxillary teeth was modeled. A 0.022" MiniSprint™ brackets and Stainless steel archwires of 0.019" × 0.025" and 0.017" × 0.025" (Forestadent, Pforzheim, Germany) were generated. Warren torque spring was modeled and used in the simulation on the upper right central incisor. Four case scenarios were simulated. In two scenarios, the archwires were untouched for both archwire sizes. In comparison, in the other two scenarios, each archwire size was rounded for the upper right maxillary incisor bracket area. Stresses in the Warren torque springs were calculated, the root tip displacement in the four scenarios was measured in millimeters, and both were analyzed.

Results: The root tip displacement was highly affected by rounding the archwire. The increase in root tip displacement was 1538% for the Warren torque spring on 0.019" × 0.025" and 783% for 0.017" × 0.025". The amount of root tip displacement was about 18.8 mm for 0.017" × 0.025" with rounding and 12.2 mm for 0.019" × 0.025". The concentration of the stresses in the Warren torque spring was in the neck of the spring next to the coils.

Conclusion: Rounding the archwires while using the Warren torque spring on a rectangular archwire will increase the efficiency of the spring and, in turn, will exhibit more torque on the tooth. Smaller dimensions of rectangular archwires will give more torque in conjunction with Warren torque springs compared to larger sizes of archwires.

## Introduction

The quest for optimal torque control in fixed orthodontic appliances continues to be a pivotal aspect of contemporary orthodontic treatment.

Achieving the proper buccolingual inclination of teeth is a cornerstone in orthodontic treatment, directly impacting the attainment of ideal occlusal relationships and long-term stability. Inadequate buccolingual inclinations, particularly in the anterior teeth, can lead to space constraints within the dental arch [1], difficulties establishing a solid class I relationship with anterior guidance, and compromised smile aesthetics. Conversely, incorrect inclinations in posterior segments pose challenges in achieving ideal cusp-to-fossa relationships between maxillary and mandibular teeth [2].

Numerous factors influence torque, a critical component in controlling buccolingual inclination. These include anatomical irregularities of teeth, the size and morphology of archwires, the way archwires engage with brackets, as well as the position, slot size, and material properties of the brackets [3-10]. To finish orthodontic cases optimally, an effective torque expression that moves the tooth in its proper position across all three planes is imperative [11].

This research introduces an innovative modification to traditional torque mechanics, particularly addressing the limitations of rectangular wires with torque springs, as suggested in the work of Burstone and Choy [12]. The modification involves strategically altering rectangular wires, rounding the wire at the bracket-engaging area while positioning the Warren torque spring on the adjacent rectangular section. This novel approach challenges the conventional belief of a "failed mechanic" by proposing that such a modification could effectively enhance torque application.

In the field of orthodontics, complex biomechanical questions like the application of orthodontic force to teeth are increasingly being assessed using the finite element (FE) method. This method has proven invaluable in numerous orthodontic studies. For instance, the FE method has been used to assess the center of resistance of teeth, a crucial aspect of understanding the biomechanical response of teeth to applied forces. It has also been instrumental in examining various aspects of orthodontic efficiency, different bracket types, anchorage methods, surgical modalities, and retention procedures.

Specifically, finite element analysis (FEA) is employed in orthodontics to analyze the biomechanical effects of treatment modalities, allowing for the calculation of deformation and stress distribution in the bodies exposed to external forces [13]. This method provides detailed and precise information regarding stress on load application, which is crucial for assessing the biomechanical aspects of orthodontic treatment [14].

The FE method in orthodontics also contributes significantly to solving structure-mechanical and biomechanical problems, such as determining the mechanical properties of the periodontal ligament and simulating tooth movements [15]. Furthermore, FEA has been used to simulate and analyze biomechanical reactions during dental displacements due to orthodontic treatments with fixed or removable appliances [16].

Thus, the FE method has become an essential tool in orthodontic research, providing a deeper understanding of the biomechanical principles underlying orthodontic treatments and aiding in the development of more effective and efficient treatment strategies. To validate this hypothesis and purely test the mechanics without the variables introduced by human biology, a 3D model of a typodont was utilized, followed by FEA. This methodological choice underscores the initial focus on mechanical efficacy, with further research on FEA of human skulls or in vivo studies suggested if the mechanics prove successful.

Recent literature underscores the ongoing evolution in torque control. Studies such as those by Migliorati et al. highlight the efficiency of the ligature-archwire-slot system in lingual orthodontics for torque control, especially with specific NiTi wires [17]. Arnold et al. demonstrate that a high level of torque control can be achieved with specific types of stainless steel wires in orthodontics [18]. Additionally, the study by Sanders et al. compares multiforce NiTi wires to multistrand wires without force zones, revealing better torque control with the former in fixed orthodontic appliances [19].

The primary focus of this research is to investigate Burstone's hypothesis about Warren torque springs when applied to rectangular wires [12]. Additionally, it examines the hypothesis of rounding these wires between the bracket wings of the target tooth to be moved with labial root torque. This study aims to determine whether the rounding of wires, in conjunction with the use of torque springs, influences orthodontic outcomes, addressing a notable gap in current literature and resolving controversies in orthodontic practice.

## Materials and methods

Geometry

**Figure 1:**
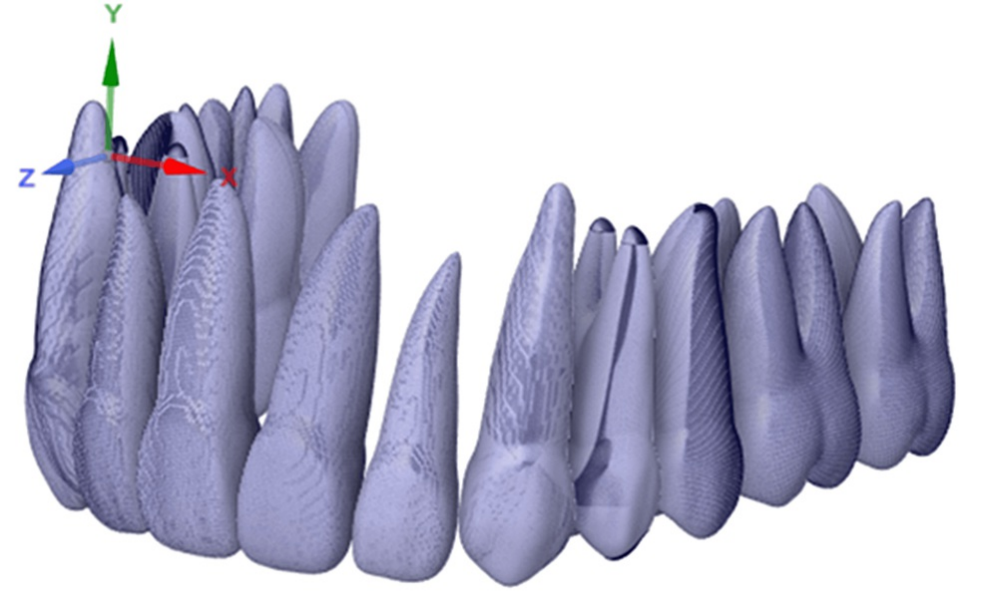
3D surface mesh data (OBJ format). Source: Authors.

The initial step for any FEA simulation is to create a geometry or computer-aided design (CAD) model. Therefore, a 3D CAD model in OBJ (object file) format was created using an available 3D set of upper teeth online [20]. The 3D model was rescaled to sizes mimicking natural teeth average dimensions, taking into account the height [21]. Then the rescaled teeth were mirrored to generate the left-side upper teeth (Figure 1).

The teeth arrangement shown in Figure 1 was random. These teeth were 3D printed using Crowntec (Saremco Dental AG, Rebstein, Switzerland) and arranged on an articulator with an occlusal convex plate, and a typodont was formed. The teeth were bonded with MiniSprint MBT brackets (Forestadent, Pforzheim, Germany) using indirect bonding trays digitally fabricated in Ortho Studio (3Shape, Copenhagen, Denmark) and 3D printed using Crowntec (Saremco Dental AG). The brackets were positioned according to the average bracket heights as suggested in the recommended bracket positioning chart [22].

**Figure 2:**
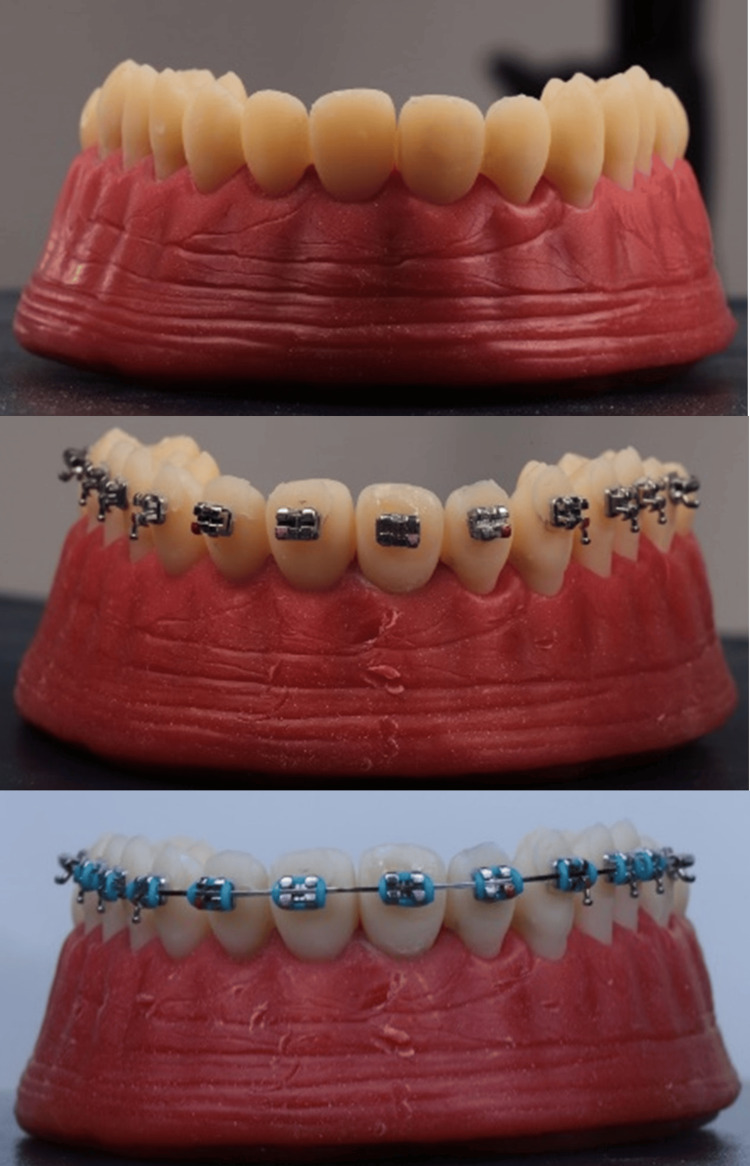
Typodonts made from 3D printed teeth of the model and underwent leveling and alignment. Source: Authors.

The typodonts underwent leveling and alignment using two archwires: 0.018" and 0.019" x 0.025" Titanol Budget (Forestadent, Pforzheim, Germany), successively to prepare the brackets to receive 0.019" x 0.025" Stainless steel archwires (Forestadent, Pforzheim, Germany) (Figure 2).

**Figure 3:**
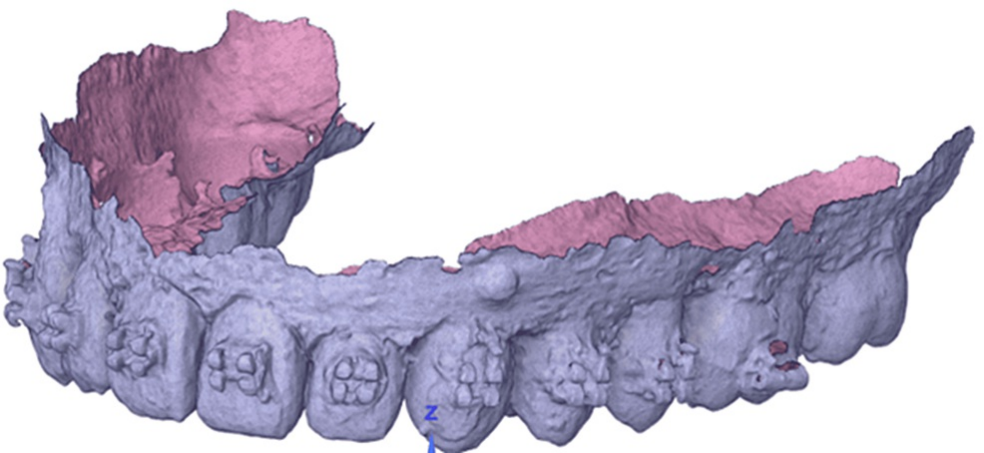
Scan data of teeth, brackets, and wax from experiments (OBJ format). Source: Authors.

Thereafter, the positions of the teeth relative to each other must be adjusted to mimic experiments. The arrangement of brackets and teeth inside wax used in experiments, scanned with Medit i700 wireless scanner (Medit, Seoul, Korea), is shown in Figure 3.

**Figure 4:**
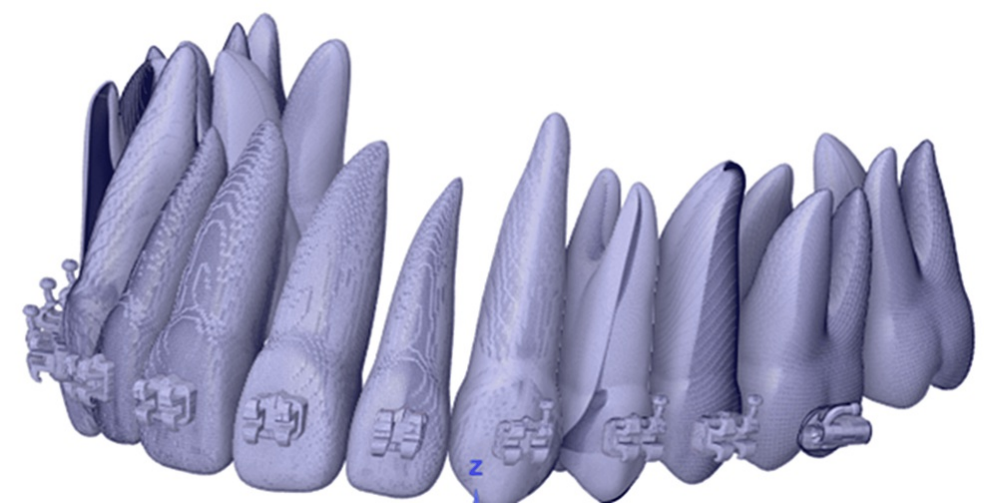
Adjusted position of brackets and teeth. Source: Authors.

The scanned data of teeth in the OBJ format has been imported into Fusion 360 software (Figure 2). Fusion 360 is favored for transforming CAD data into mesh for simulation in Abaqus due to its efficient parametric design capabilities and the ability to produce detailed 3D models, which is essential for creating precise meshes from complex geometries necessary for accurate Abaqus simulations [23]. Available teeth models are manually superimposed onto this scan data model to position as per experiments (Figure 1). A similar procedure for brackets has been followed. The adjusted teeth and brackets are shown in Figure 4, which will be processed further to perform FEA.

It is necessary to convert STL data (surface mesh) into a 3D solid CAD model, i.e., STEP format. As FEA works more efficiently with 3D CAD models, this has been accomplished using the advanced features available in Fusion 360. FEA was then performed on this converted solid 3D CAD model. After that, this model was converted from the surface mesh to .STEP file.

FEA model setup

A 3D solid CAD model from Fusion 360 has been imported into Abaqus (shown in Figure 4) for performing tooth movement simulation. Abaqus is highly regarded in the fields of engineering and dentistry for its effective simulation of material behaviors, essential in FEA studies [24]. Simulation has been performed on a model with both square and round cross-section (c/s) archwires at the area between the coils of the torque spring. The analysis cases (ACs) to study different wire setups are as follows: AC1: Rectangular c/s archwire: 0.019" x 0.025", AC2: Rectangular c/s archwire: 0.017" x 0.025", AC3: Rectangular c/s archwire 0.019" x 0.025", rounded 0.019" diameter near bracket of the tooth to be moved, and AC4: Rectangular c/s archwire 0.017" x 0.025", rounded 0.017" diameter near bracket of the tooth to be moved.

**Figure 5:**
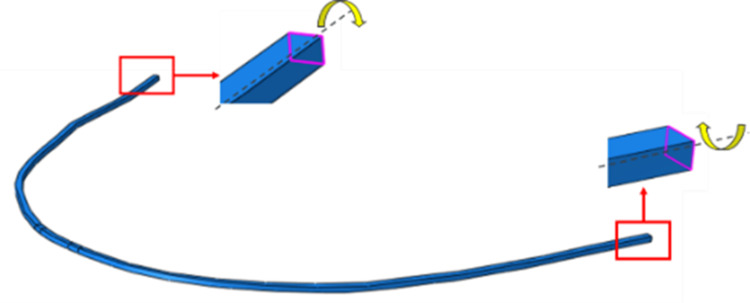
Typical setup of rectangular archwire. Source: Authors.

**Figure 6:**
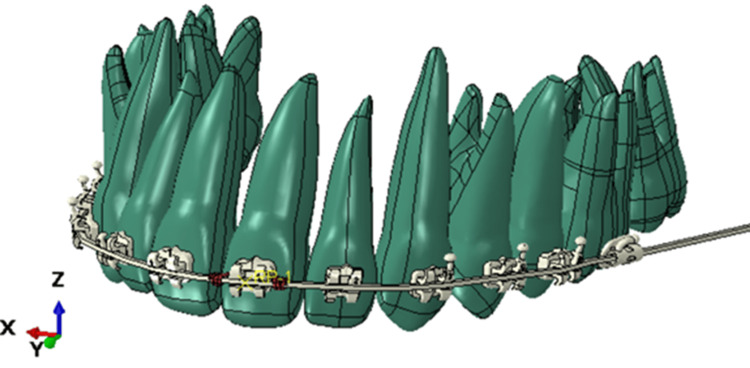
3D solid model (STEP format) imported into Abaqus. Source: Authors.

Wax has not been considered in FEA. As the modulus of wax (approximately 0.1-1.0 MPa) is negligible compared to others, such as teeth and steel wires/brackets, it offers less resistance to tooth movement. The purpose of wax in typodont experiments is primarily to hold the tooth in place after cooling down. A typical setup of rectangular c/s archwire is shown in Figure 5, and a setup of the solid CAD model is shown in Figure 6.

Meshing

In this study, a CAD model was developed, employing a meshing process with tetrahedral (TET) elements using a free meshing technique and the default algorithm in Abaqus/CAE. The spring and UR1 (the tooth intended to be displaced by spring action) were meshed with C3D10 elements, which are second-order TET elements. Meanwhile, the other teeth, brackets, and archwire were meshed using C3D4 elements, first-order TET elements. The complete model comprises 312,980 elements and 96,396 nodes, providing a detailed and comprehensive representation for analysis.

**Figure 7:**
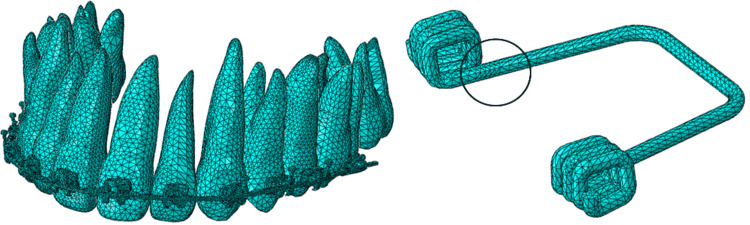
Optimum FEA mesh of complete orthodontic setup in Abaqus. FEA: finite element analysis. Source: Authors.

Since teeth, brackets, and archwires do not generate high stresses/strains, they are meshed with first-order TET elements. Their mesh size does not have a significant effect on tooth displacements. The roots of the spring undergo significant stresses and plastic strains, contributing to the spring action force and, thereby, tooth displacement. Therefore, the spring is meshed with second-order TET elements and spring roots (highlighted in Figure 7) with relatively finer mesh sizes to capture plastic strains.

The mesh size, as described above, is optimum and is a trade-off (neither too coarse nor too fine) between the accuracy of the results and FEA solver performance. Very coarse mesh can lead to inaccurate results, while too fine mesh results in extended analysis run time. Therefore, the mesh convergence study has been performed to determine the optimum mesh size to analyze AC3 (Table 1). Since spring pre-load action and mesh size have a greater impact on tooth tip displacement, the study has been performed on two levels of mesh refinement in spring. The remaining setup, such as tooth, brackets, and archwires, has been studied with Level 1 of refinement. The optimum mesh size thus obtained (shown in Table 1 and depicted in Figure 7) has been used for all ACs.

**Table 1:** Results of mesh convergence study performed with different mesh sizes for AC3. AC: analysis case.

	Spring mesh size (mm)	Archwire mesh size (mm)	Tooth UR1 mesh size (mm)	Bracket UR1 mesh size (mm)	UR1 tip displacement (mm)
Optimum mesh	0.1	0.35	1.0	0.85	12.26
Mesh refinement (Level 1)	0.05	0.2	0.5	0.45	12.28
Mesh refinement (Level 2)	0.025	0.2	0.5	0.45	12.33

Material properties

The mechanical material properties of different setups considered for FEA are listed in Table 2 [25,26]. Elastic properties were taken into account for brackets, archwire, and teeth.

**Table 2:** Material properties of different FEA components. FEA: finite element analysis.

	Steel (brackets, archwire, spring)	Saremco Crowntec resin photopolymer (teeth)
Elastic modulus (MPa)	210,000	4,000
Poisson's ratio	0.3	0.35

However, the spring undergoes plastic deformation during pre-load. Therefore, elastic-plastic properties need to be defined. Elastic-plastic properties are not known and, therefore, have been assumed (Table 3).

**Table 3:** Elastic-plastic properties of spring (yield stress in MPa).

Yield stress	Plastic strain
12,000	0
18,000	1

Analysis setup

FEA has been carried out in two steps: Step 1: Spring pre-load ~80 degrees rotation and Step 2: Spring force acting on tooth.

Interactions and boundary conditions

Interactions for all ACs (AC1, AC2, AC3, and AC4) are comprehensively defined. These include bonded contact between teeth and brackets, spring-archwire interaction through bonded contact, and two types of frictional contacts: one between the archwire and brackets with a coefficient of friction of 0.1 and another between the spring and tooth with a coefficient of friction of 0.1. Similarly, boundary conditions for these ACs are specified. Teeth are constrained initially in Step 1 and are then allowed to move freely in Step 2. As detailed in Figure 6, the spring faces undergo a rotation of 80 degrees about the X-axis in Step 1 and are free to spring back, exerting force on the tooth in Step 2. Additionally, the ends of the archwire are permitted free rotation about an axis (highlighted in the same figure) while being constrained in all other degrees of freedom (DOFs) to avoid displacement from their intended positions.

**Figure 8:**
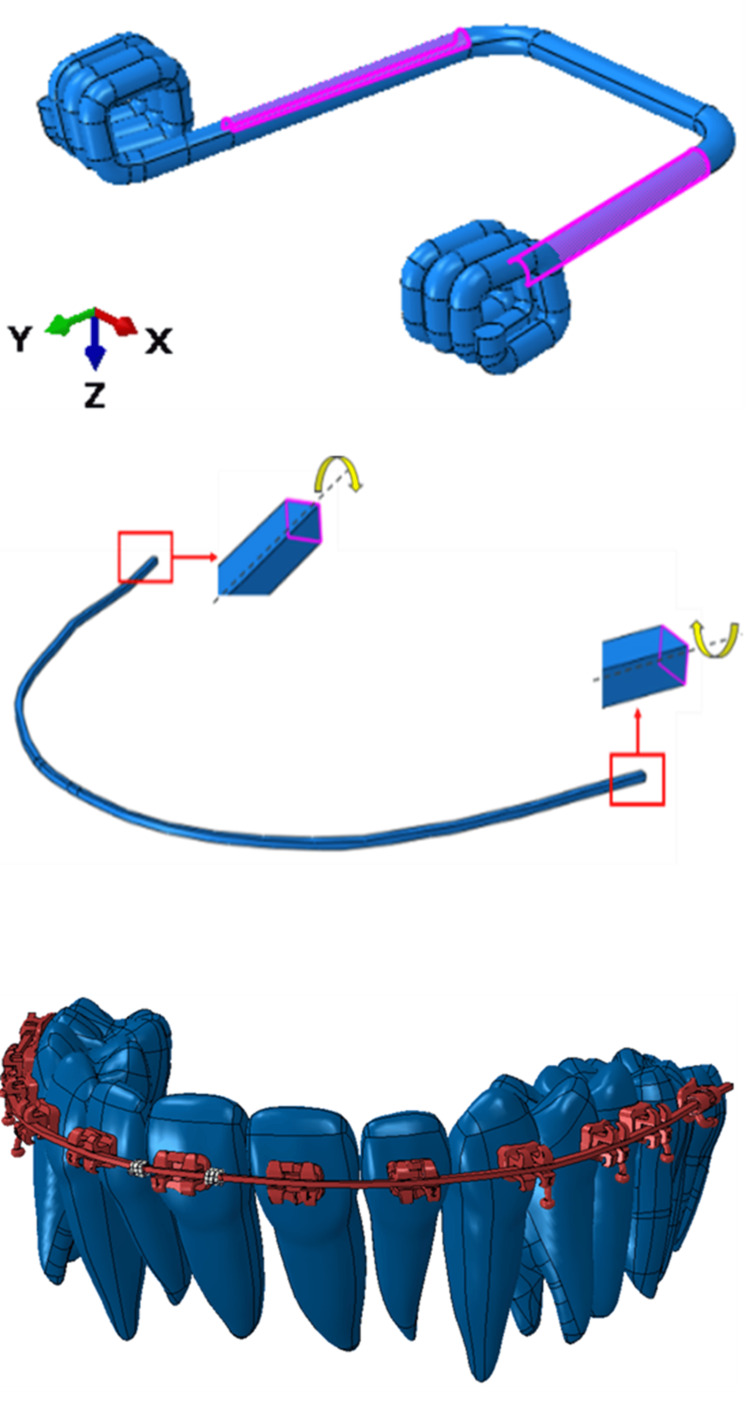
Different boundary conditions. Source: Authors.

Specifically for AC3 and AC4, the round cross-section (c/s) wire has not been physically modeled. Instead, roundness is considered with the faces of the archwire and bracket (which are supposed to be round) being constrained in all DOFs except for rotational DOF, simulating a hinge-like connection, as shown in Figure 8.

## Results

**Figure 9:**
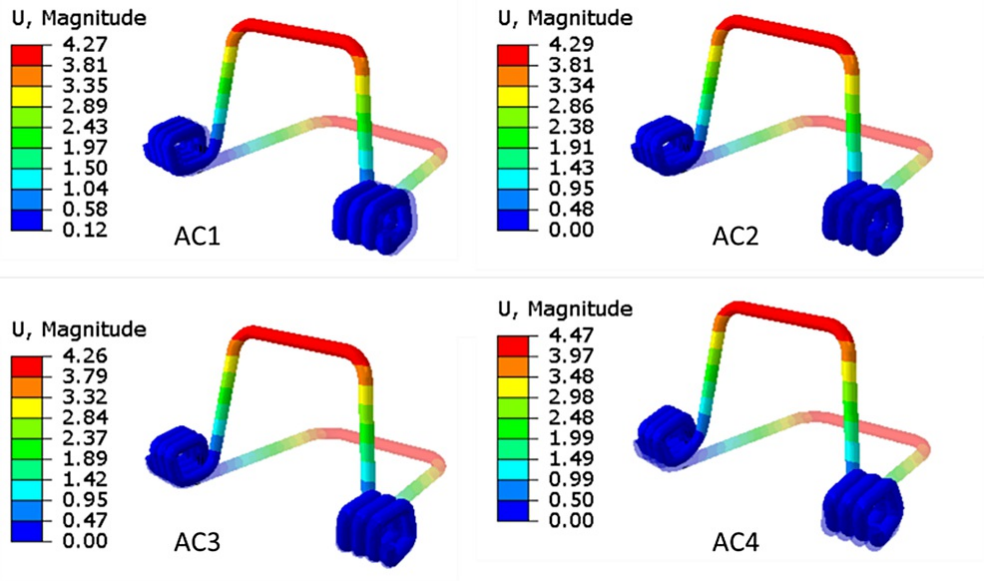
Contour plots of spring displacement (mm) under the application of spring pre-load (Step 1) for all ACs (AC1, AC2, AC3, AC4). AC: analysis case. Source: Authors.

**Figure 10:**
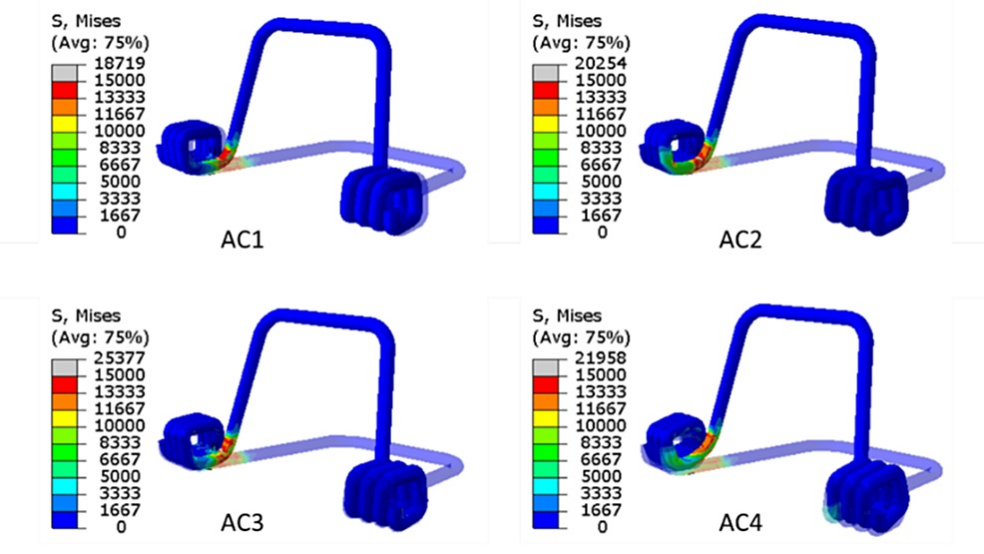
Contour plots of von Mises stress (MPa) in spring under the application of spring pre-load (Step 1) for all ACs (AC1, AC2, AC3, AC4). AC: analysis case. Source: authors.

Step 1 results for spring pre-load for all ACs (AC1, AC2, AC3, AC4) have been presented. The displacement and stress results in Step 1 are shown in Figure 9 and Figure 10, respectively. All ACs have been given pre-defined spring pre-load displacements and confirmed by the results. The amount of pre-load and the stress distribution pattern are the same for all the analyses. It shows that the maximum stress accumulates in the spring's neck.

**Figure 11:**
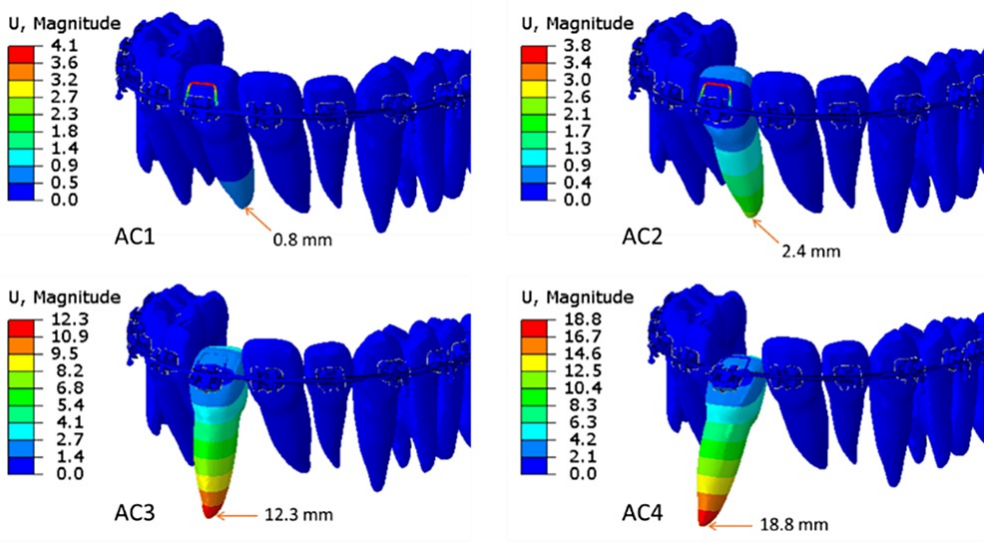
Contour plots of tooth tip displacement and maximum tooth displacement under the application of the spring release (Step 2) for all ACs (AC1, AC2, AC3, AC4). AC: analysis case. Source: Authors.

Step 2 results, i.e., pre-loaded spring exerting forces on the tooth and thereby movement, are presented in Figure 11. Note that tooth movement in AC1 and AC2, i.e., complete rectangular c/s dimensions, is less than in AC3 and AC4, where the engaging part into the bracket slot is rounded.

Displacement results from the simulation presented in Figure 11 align with these mechanics. The tooth movement magnitudes from the simulation agree with test results done on the typodonts, as shown in Table 4.

**Table 4:** Root (UR1) tip displacement. AC: analysis case.

AC	Simulated tooth tip displacement, UR1 (mm)	Experimental tooth tip displacement, UR1 (mm)
AC1	0.8	1.5
AC2	2.4	3.0
AC3	12.3	9.0
AC4	18.8	18.5

The results show that with the rounding of archwires in between the brackets of the tooth to be moved, the root tip movement increased from 0.8 mm to 12.3 mm for 0.019" x 0.025" stainless steel wires. For 0.017" x 0.025" stainless steel wires, the root tip displacement increased even more, from 2.4 mm to 18.8 mm.

The results show an increase of 1538% of root tip displacement buccally for rounding the cross-section of the archwire 0.019" x 0.025" between the wings of the brackets of UR1, while there is an increase of 783% of root tip displacement for the 0.017" x 0.025" archwire (Table 5).

**Table 5:** Root tip displacement increase (%) after archwire rounding between bracket wings. c/s: cross-section.

Archwire	Amount of movement (mm)		Displacement difference in mm	Increase displacement by %
	Rectangular c/s	Rounded c/s		
0.019" x 0.025"	0.8	12.3	11.5	1538
0.017" x 0.025"	2.4	18.8	16.4	783

## Discussion

This research comprehensively analyzed the torque exerted on a single tooth, mainly focusing on the root tip displacement. This was achieved by using a Warren torque spring on rectangular archwires. Subsequently, a comparative study was undertaken where the same spring was utilized on identical archwires. However, in this experiment phase, we introduced a significant modification: the part of the archwire between the spring's coils, which interacts with the bracket wings on the target tooth, was rounded. This modification aimed to eliminate potential engagement between the bracket and the archwire within the specified region. In this in silico study, we investigated the torque effect of a Warren torque spring on a single tooth, with a specific focus on root tip displacement, using rectangular archwires. Additionally, we assessed von Mises stress in the springs to pinpoint areas of maximum stress. This comprehensive analysis aids in understanding the biomechanical behavior and optimizing orthodontic treatments.

The von Mises stress, widely utilized in FEAs, proves effective as it amalgamates principal stresses into a single equivalent stress. This equivalent stress is akin to the yield stress, providing a more accurate prediction of potential system failure [27]. It was observed that the root tip displacement is highly affected by rounding the wire between the wings of the bracket of the tooth to be moved with the torque spring.

The finite element method (FEM) is a crucial tool in orthodontics for biomechanical simulations, aiding in the understanding of the behavior of biological structures in various scenarios. Although it yields numerical approximations, FEM is invaluable in clinical research when direct in vivo measurements are challenging [28-32]. These insights from FEM significantly enhance orthodontic treatment planning and outcomes.

The strength of this study is the validation of the results by comparing them with the results of the experiment done on the typodont, and they agreed. This is more accurate than other studies that do not validate the results with real-life experiments [11,27,33,34].

The findings of this study align with Burstone's theoretical proposition that employing auxiliary torque springs in orthodontics represents impossible biomechanics, but using the same spring on a round wire makes the mechanics possible [12]. Comparing these results with other research is challenging due to the scarcity of studies specifically focused on Warren torque springs in orthodontics or any similar auxiliary springs serving the same purpose [35-37].

The effectiveness of the Warren torque spring on rectangular wires is notably constrained. This is primarily because the rectangular archwire fits tightly inside the bracket, controlling tooth movement. The larger the archwire's dimensions, the less movement is observed. By rounding the wire, root displacement of the teeth is enhanced, as this modification eliminates any engagement between the archwire and the bracket slot. Per Newton's third law, which states that every action has an equal and opposite reaction, the loading of the torque spring generates a reactive force on the archwire, causing it to twist in the direction opposite to that required for tooth torque. Consequently, the action of the Warren torque spring and the reactive twisting of the archwire negate each other, resulting in a state of equilibrium.

Using Warren torque springs on rectangular archwires resulted in some tooth movement, although the extent varied based on the dimensions of the archwire. The difference in movement between teeth on 0.019" x 0.025" archwires compared to 0.017" x 0.025" archwires without rounding is due to the amount of play between the archwire and the bracket slot. More play allows more room to torque the tooth before the archwire fully engages with the bracket slot. This explains why the Warren torque spring on the 0.017" x 0.025" archwire resulted in more root tip displacement than the 0.019" x 0.025" archwire.

The innovative approach of shaping the archwire to round off between the bracket wings, such that the clamps of the Warren torque spring rest on the wire's rectangular section while the rounded portion is engaged between the bracket wings, significantly boosted the effectiveness, showing a remarkable 1538% increase in root tip displacement. This enhancement was more pronounced with the 0.019" x 0.025" archwire (1538%) compared to the 0.017" x 0.025" archwire (783%). This more significant difference with the 0.019" x 0.025" archwire is because of its fuller engagement within the bracket, which restricts tooth movement more when using the Warren torque spring.

Interestingly, the displacement of the root tip was more significant with the 0.017" x 0.025" archwire compared to the 0.019" x 0.025" one, measuring 18.8 mm for the former and only 12.3 mm for the latter. This discrepancy is attributed to the von Mises stress distribution observed in the initial phase of the FEA. The peak stress was noted at the junction of the Warren torque spring adjacent to its coils. Due to the rectangular formation of the coils for engagement on the archwire, they lose their inherent coiling function and essentially become clamps. Consequently, the stress levels surpass the elastic limit of the stainless steel spring, leading to its deformation during the initial loading phase. The difference in deformation is primarily because the 0.017" x 0.025" archwire, being more pliable, allows more significant twisting when pre-loaded. This flexibility reduces deformation, and as the spring exerts force on the tooth (diminishing the counterforce on the archwire), the archwire reverts to its original position. This action further triggers the spring, resulting in additional root tip movement.

However, a fundamental limitation of this research is that it was conducted on typodonts instead of anatomical models. The use of typodonts as substitutes for actual human anatomical models in dental research carries significant limitations. Typodonts, designed for mechanical simulation, fail to encapsulate the biological complexities of human oral tissues, notably the viscoelastic properties of the periodontal ligament and the biological responsiveness of alveolar bone. This results in a disparity between simulated outcomes and actual clinical scenarios, as typodonts cannot mimic biological reactions such as tissue inflammation and remodeling. Additionally, typodonts lack representation of the diversity in root morphologies and individual variances found in patient populations, elements that are crucial in orthodontics [38].

Moreover, FEMs using typodonts are limited by predefined numerical values and fail to accommodate the nonlinear, time-dependent nature of biological tissues under orthodontic forces. The static nature of these simulations overlooks the dynamic physiological changes occurring during treatment, including the remodeling of the periodontium and biological responses to stress. Consequently, there is a lack of consideration for factors like biochemical responses of periodontal ligament cells to mechanical stress and the influence of cyclic masticatory forces. This leads to a significant gap in accurately predicting tooth movement, necessitating future research to integrate biological variables into simulation models for a more realistic representation [39,40]. Advanced modeling techniques are necessary to capture the dynamic and biological complexities of orthodontic tooth movement.

## Conclusions

From this in silico study, several conclusions can be drawn: Firstly, rounding the archwire between the coils of the torque spring, which engages with the bracket wings on the tooth to be moved, increases the spring's efficiency in torquing the tooth. Secondly, smaller rectangular archwire sizes paired with Warren torque springs exert more torque on the tooth, irrespective of the wire being rounded. Lastly, peak stress tends to accumulate in the neck of the torque spring, leading to plastic deformation due to reduced resiliency, as the coils primarily function as clamps.
